# Surgical Dynamometer to Simultaneously Measure the Tension Forces and the Distance between Wound Edges during the Closure of a Laparotomy

**DOI:** 10.3390/s18010189

**Published:** 2018-01-11

**Authors:** Joan Roca, Miquel Nogués, Rafael Villalobos, María Carmen Mías, Martí Comellas, Cristina Gas, Jorge Juan Olsina

**Affiliations:** 1 Department of Computer Science and Industrial Engineering. University of Lleida—Escola Politecnica Superior. C. Jaume II, 69, E-25001 Lleida, Spain; jroca@diei.udl.cat (J.R.); mnogues@diei.udl.cat (M.N.); 2 Surgical Department—Arnau de Vilanova University Hospital. Av. de l’Alcalde Rovira Roure, 80, E-25198 Lleida, Spain; rafovilla26@gmail.com (R.V.); maricarmen.mias@gmail.com (M.C.M.); crisgas6@gmail.com (C.G.); jjolsina@gmail.com (J.J.O.)

**Keywords:** dynamometer, surgery, closing forces, laparotomy, incision separation

## Abstract

The closure of the abdominal wall after making a laparotomy is a major challenge for surgeons, since a significant percentage of closures fail and incisional hernias rise. The suture has to withstand the forces required to close the incision, while not hindering the adequate wound healing progression. Currently, there is no surgical measuring device that could be used to determine the required closing forces, which can be very different depending on the patient. This paper presents a dynamometer to measure the tension forces to be applied while closing a surgical incision, and it simultaneously measures the distance between wound edges. It is a compass-like instrument. A mechanism between the two legs incorporates a load cell, whose signal is read by an electronic device that computes the values of the tension forces between wound edges. An angular position sensor at the pin joint between legs provides the distance between both sides of the incision. Measuring capabilities of the instrument prototype were verified at the laboratory. Thereafter, its functionality was demonstrated in experimental surgery tests. Therefore, the instrument could be very useful in clinical applications, assisting personalized surgical techniques.

## 1. Introduction

Wound healing of any surgical incision is a complex process in which several biomechanical and biochemical disorders can appear. In particular, the closure of the abdominal wall after making a midline laparotomy is a major challenge to any surgeon, since a defect may occur after this closure, giving rise to an incisional hernia.

The percentage of such cases after a laparotomy can be up to 15–20% within some years after surgery [1,2].

Surgical suture has to stand the tension forces required to maintain the incision wound edges touching each other until the wound healing process provides sufficient strength to the injured tissue. Despite this, the strength of it will never achieve the one prior to surgery [3].

**Figure 1:**
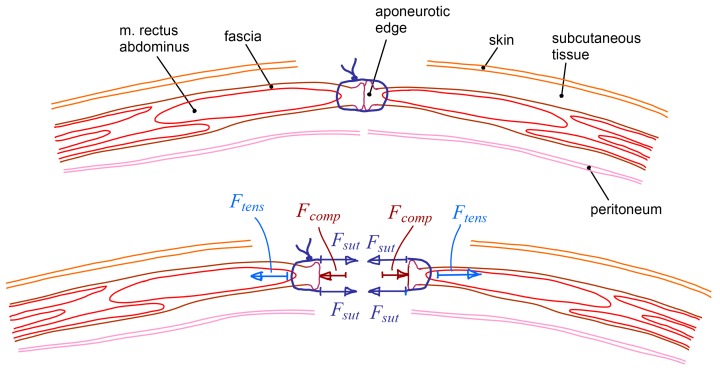
Common laparotomy closure and its acting forces.

Figure 1 shows a common laparotomy closure with a surgical suture. It also shows the different forces acting due to the abdominal wall closure.

The forces exerted by the suture threads (*∑F_sut_*) have to compensate the tension force at the aponeurotic edge (*F_tens_*) and are also responsible for the compression force between wound edges (*F_comp_*). Then, the relationship between such forces is expressed according to Equation (1).

(1) $$\sum F_{sut}=F_{tens}+F_{comp}$$

The suture has to ensure that wound edges keep touching each other, but the compression force between them should be minimal to avoid irrigation problems that could affect wound healing. Thus, the forces exerted by the suture threads should be slightly higher than the tension force at the aponeurotic edge. On the other hand, the forces exerted by the suture threads should be limited to prevent suture failure that may occur by thread breakage or, most likely, by tissue tearing. Usually, tension forces along the aponeurotic edge are not measured during the closure of a laparotomy. However, it is known that they can be very different depending on the patient, the area of the incision and the neuromuscular relaxation [4]. In 1990, Ramirez introduced the “components separation technique” to reduce the tension force at the aponeurotic edges before the abdominal wall closure [5]. It can be an adequate procedure in cases where the tension forces are too high or when the aponeurotic tissue resistance is lower than expected. Nonetheless, this technique also has some drawbacks and should not be applied unless absolutely necessary [6,7,8].

Another common approach to prevent dehiscence after the closing of a laparotomy is the use of mesh augmentation. Nevertheless, this technique should only be applied when required, since it also has some disadvantages and it increases both the cost and required time for the surgical operation [9]. Currently, the application of such techniques and other aspects of the laparotomy closing procedure are decided based only on surgeon experience and clinical approach, since there is no clinical or surgical measuring device that can be used to determine the closing forces.

There are some studies about tension forces to close a laparotomy in cadavers [10]. They used a standard spring dynamometer to apply a traction force to one side edge of the cut tissue. Thus, tissues can move laterally and then the measured forces are only partly related to the ones that would be present when the edges approach symmetrically. The distance that the edge has advanced is measured using a caliper, hence it is quite difficult to do it simultaneously to the dynamometer measurement.

Horeman et al. [11] developed a set of two sensors to measure the forces in a suture: the stitch force and the thread end pulling force. Those are very interesting and useful devices to evaluate and to check the resulting stitch force in a suture.

The aim of this work is to develop a dynamometer to measure the required transverse tension forces to close a particular area along the incision of a laparotomy. The instrument should also be able to measure the necessary force to maintain a certain distance between wound edges, while closing the incision, and simultaneously measuring the distance between these edges.

## 2. Materials and Methods

### 2.1. Instrument Mechanical Design

Force and distance requirements must be established before addressing instrument mechanical design. According to previous studies [4,7], and some initial trials carried out by the authors [12], maximum transverse tension forces at different areas of the laparotomy in human patients would be in the range of 2–15 N. Maximum distance between wound edges, before the abdominal wall closure, would be about 100 mm. Thus, measurement range for the new instrument has been established in 0–20 N for tension force and 0–130 mm for distance. The desired accuracies for the measurements have been established in ±0.2 N for force (±1% FS) and ±2 mm for distance (±1.5% FS), which are thought to be more than sufficient for such application.

**Figure 2:**
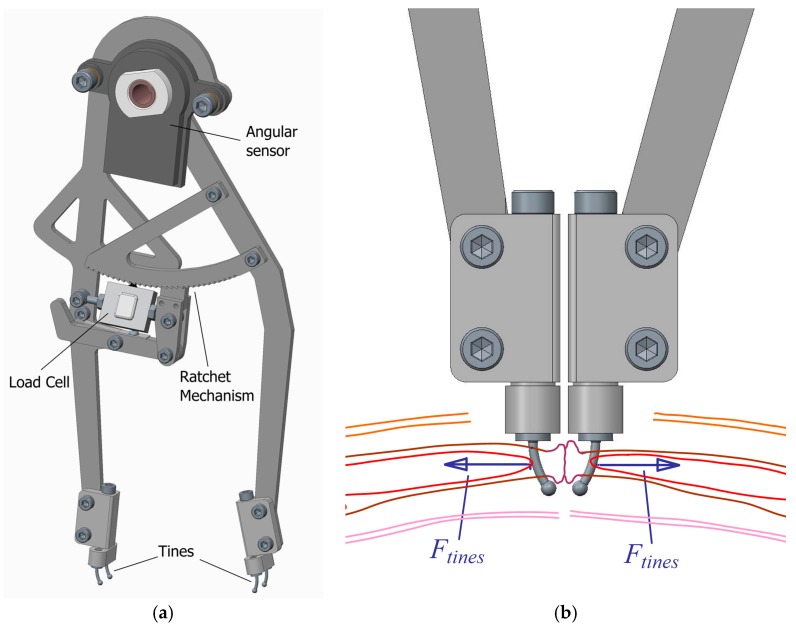
Instrument: (**a**) Conceptual design; (**b**) Forces applied by the aponeurotic edges to the tines.

Once requirements are established, the mechanical design can be defined. A compass-like instrument is proposed, with two articulated legs that can bring closer or move away their ends (Figure 2a). At each end, there is a set of two small tines to hold an area of the aponeurotic edge of the tissue that was previously incised. Then, the instrument measures the force applied to both sides of the incision area to maintain a certain distance between the edges (*F_tines_*) (Figure 2b).

**Figure 3:**
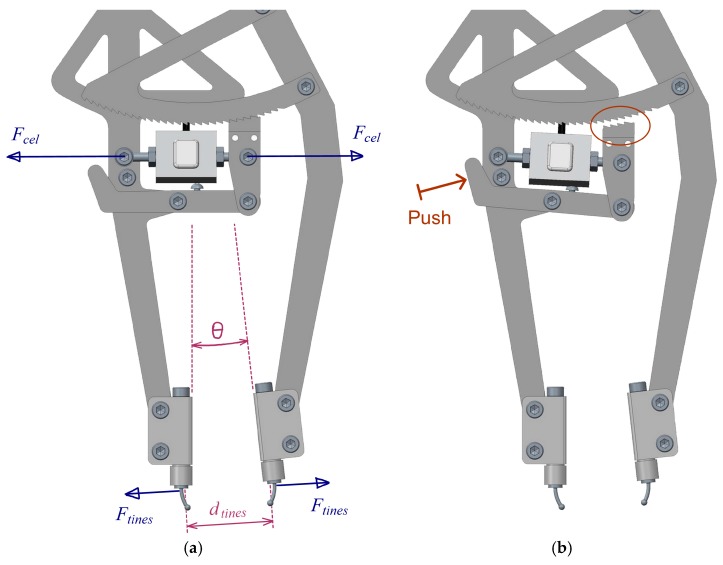
(**a**) Main variables involved in the measuring procedure; (**b**) Ratchet mechanism disengaged while not measuring, to separate the ends of the legs.

A ratchet mechanism linking the two legs is intended to lock them in different angular positions, corresponding to different distances between edges. The increment of such distance between any pair of consecutive locked positions is approximately 5 mm. The mechanism incorporates a load cell and its signal is read by an electronic device, which after making the appropriate operations, depending on the geometry of the mechanism, computes the value of the separating forces at the tines (*F_tines_*) (Equation (2) and Figure 3a).

(2) $$F_{tines}=\frac{0.2197}{\cos\left(\frac{\theta }{2}\right)}\cdot F_{cel}$$

There is also an angular position sensor at the pin joint between legs that provides, through the necessary geometric calculations, the distance between tines at both sides of the incision (*d_tines_*) (Equation (3) and Figure 3a).

(3) $$d_{tines}\left(\mathrm{mm}\right)=392\cdot \sin\left(\frac{\theta }{2}\right)+8\cdot \cos\left(\frac{\theta }{2}\right)$$

Measurements, both force and distance, are simultaneously taken, and then the relationship between them can be established.

When the surgeon wants to decrease the distance, he has to push the outer sides of the legs until changing the teeth in contact in the ratchet mechanism. To increase the distance, the teeth in the ratchet mechanism have to be disengaged by pushing the balancing lever (Figure 3b).

### 2.2. Electronic Hardware and Software

As mentioned before, the instrument measures the separation force through a load cell and the distance between tines through an angular position sensor. In both cases, commercially available sensors have been used. As any surgical device, the instrument has to be sterilized. Nowadays, there are several available cold sterilization methods that operate at temperatures below 65 °C. For this reason, the specified operating temperature range for the sensors has been set between 0 °C and 70 °C. Moreover, as some of such sterilization methods consist in the immersion of the device in liquid or in a highly moisturized ambient, the minimum rate of protection specified for the selection of the sensors was IP67.

The selected force sensor is a submersible miniature s-beam load cell, made of stainless steel and with a rate of protection IP68. Its weight is only 15 g. Although the measuring range is 111 N, it has a safe overload factor of 1000% R.O. which ensures its reliability. The sensibility output value after calibration was 1.4528 mV/V using an excitation bridge voltage of 5 V DC. Under these conditions, it is achieved a non-linearity error lower than 0.05% R.O.

In order to measure the angle between legs of the instrument, a non-contacting magnetic angular sensor has been considered. Such sensor evaluates the measured angle every 1.5 ms at most, and the last calculated angle is hold to be read through an SPI communication protocol. Main sensor characteristics are 135° measurement range, a non-linearity error lower than 0.5%, a weight of 40 g, and an IP67 rate of protection.

**Figure 4:**
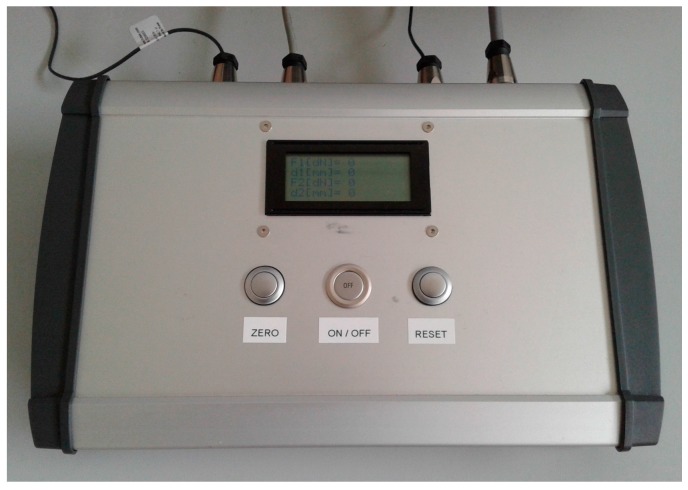
Electronic device for signal conditioning and data processing.

An electronic board has been designed and manufactured in order to get the signal from the load cell using a standard strain gauge amplifier with the corresponding signal conditioning circuit. A microcontroller acquires the conditioned signal from the load cell and the signal from the angular sensor and it computes them to obtain the desired values, which are the force acting on the tines and the distance between them. Other features are that it is feasible to simultaneously connect up to two instruments, and that all the evaluated engineering values are shown in an LCD display and can be sent to a computer through an USB port for further analysis, diagnostics considerations, or individual patient data registration (Figure 4).

### 2.3. Instrument Prototypes

**Figure 5:**
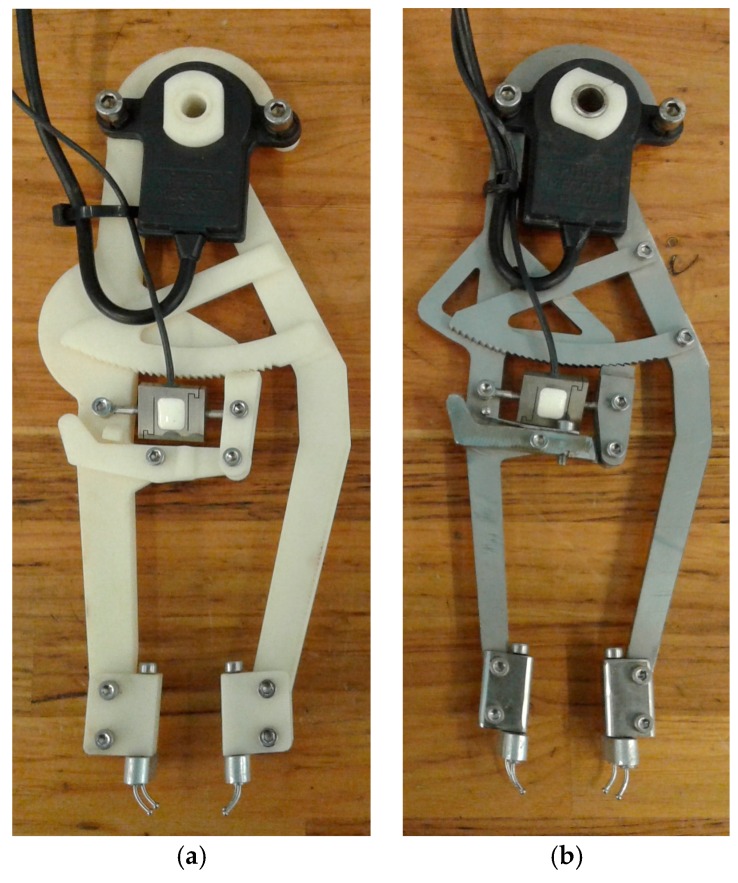
Prototypes of the instrument built: (**a**) 3D printed plastic; (**b**) stainless steel.

Some prototypes of the instrument have been built to check its functionality and to verify the accuracy of the measurements. A first version of the instrument prototype was built through rapid prototyping using 3D printed plastic (Figure 5a). The second version was built in stainless steel (Figure 5b). In both prototypes, tines are made of stainless steel, with a circular section 1.2 mm in diameter and a spherical endpoint 2 mm in diameter.

The plastic prototype allowed for checking the proper functionality of the instrument mechanisms. However, this version had the problem of poor resistance and the legs broke after several high tension measurements. This version had also the disadvantage of low stiffness, affecting the accuracy of the distance measurement in case of high tension forces. The stainless steel version of the instrument weights 286 g. In this case, the strength of the legs is more than enough. The rigidity enhancement is also very significant. However, there is a certain deformation when the applied separation force is increased and it must be taken into account to get the correct measurement of the distance between tines.

### 2.4. Verification of the Measurement System

**Figure 6:**
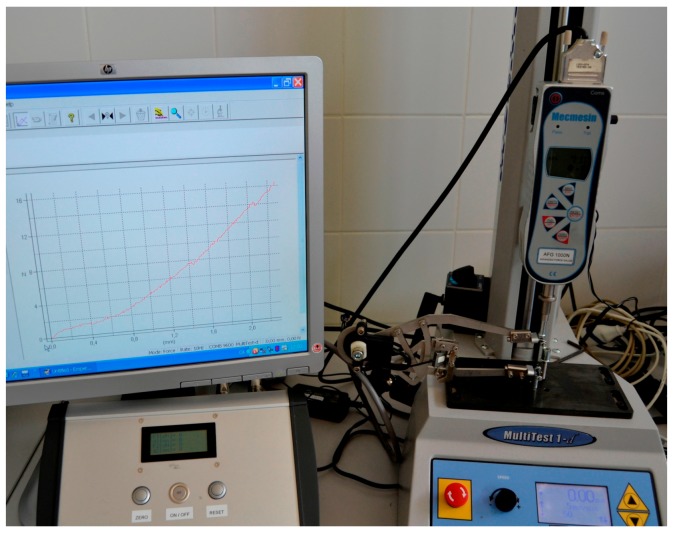
Verification test setup for force and distance deviation.

The stainless steel instrument prototype has been tested in conjunction with the electronic device, in order to verify its accuracy. Force verification tests were carried out using a standard traction/compression test bench machine (Figure 6).

During the tests, the ratchet mechanism position is kept fixed. Force verification tests also evaluate the increment of the distance between tines, due to the deformation of the instrument, when the applied force is increased.

Moreover, distance verification tests were carried out that compared the measurements using the instrument to the values obtained with a digital caliper. In this case, measurements were carried out ensuring a separating force of 0.2 to 0.4 N, in order to avoid the influence of the instrument deformation.

### 2.5. Measuring Procedure in Experimental Surgery

**Figure 7:**
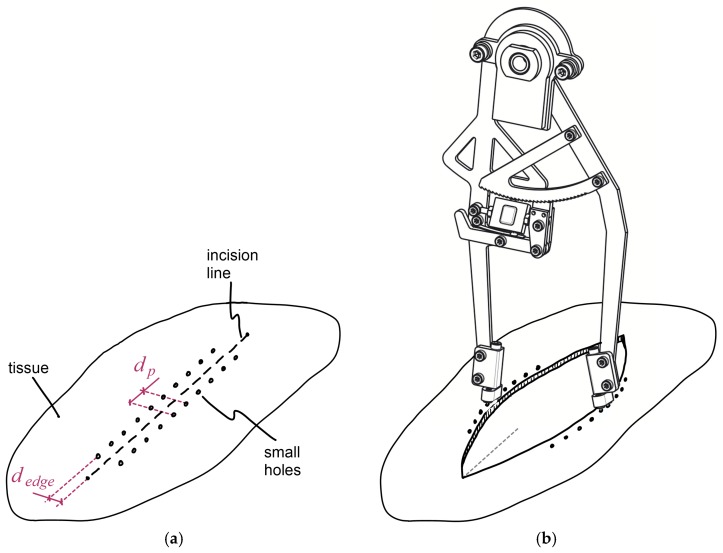
Measuring procedure: (**a**) Marks on the tissue that are required for the measuring process; (**b**) instrument linked to the tissue while measuring.

Surgical operations and measuring procedures should follow an adequate process. First of all, a line and a set of dots are marked on the tissue to be cut, in order to define the length of the incision and the positions where small holes have to be put—these will be used to introduce the tines of the instrument. They must be at a constant transverse distance *d_edge_* from the incision line and longitudinally equidistant from each other a distance *d_p_* equal to the distance between each pair of tines (Figure 7a). Subsequently, the surgeons make the incision and they perform the surgical intervention as usual. Then, before closing the main incision, it is time to carry out the measurements (Figure 7b)).

The sets of tines are inserted into the holes on the tissue that correspond to the area of the incision in which the measurements are going to be done, ensuring that both sets are symmetrically located at both sides of the incision. Surgeons can start the measurements at the situation where the distance between opposite edges is maximum, and then he can press the outer sides of the legs to a new measurement position that corresponds to a lower distance between tissue edges. The new position can be one or a few ratchet mechanism teeth away from the previous one. Once the surgeon releases the forces on the legs and the ratchet mechanism is locked, the new measurement values can be noted. The previous step can be repeated several times, for decreasing distances between edges, until they make contact.

The distance between opposite tines can even be lowered down from the previous situation. Then, the dynamometer measures the sum of the tension force at the aponeurotic edge (*F_tens_*) and the compression force between wound edges (*F_comp_*) (Figure 1). At that point, the force measured by the instrument is representative of the forces that the suture threads should exert (*F_sut_*) to hold the opposite tissue edges compressed one against the other. After that, the surgeon can disengage the ratchet mechanism and let the distance between instrument legs increase up to the initial situation. The measuring process can now be repeated for other areas of the incision.

**Figure 8:**
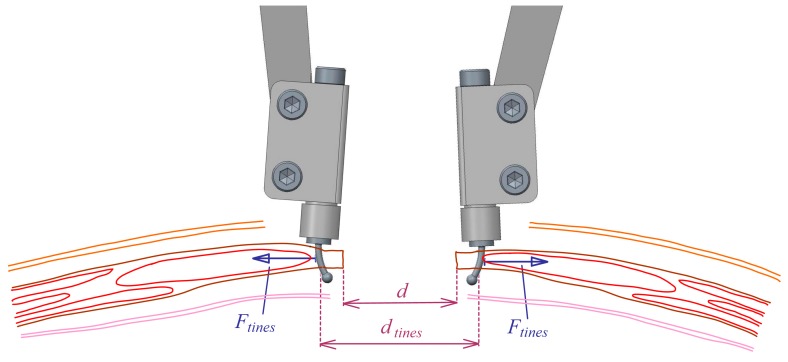
Distance between wound edges (*d*) and between tines of the instrument (*d_tines_*).

The distance between wound edges *d* (see Figure 8) can be derived from the measured distance between tines, *d_tines_*, according to Equation (4).

(4) $$d=d_{tines}-2\cdot d_{edge}$$

Ethical approval: all applicable international, national, and/or institutional guidelines for the care and use of animals were followed. All procedures performed in studies involving animals were in accordance with the ethical standards of the institution or practice at which the studies were conducted.

All protocols were submitted to the Animal Experimentation Ethics Committee (CEEA) at the University of Lleida. This committee ensures that the experimental design is in line with current legislation, collaborates with researchers to analyze the ethical implications of projects, and draws up reports for funding applications, when required. The authorization of a local ethics committee is compulsory to initiate any research activity raising ethical issues.

## 3. Results

### 3.1. Verification of the Measurement System

**Figure 9:**
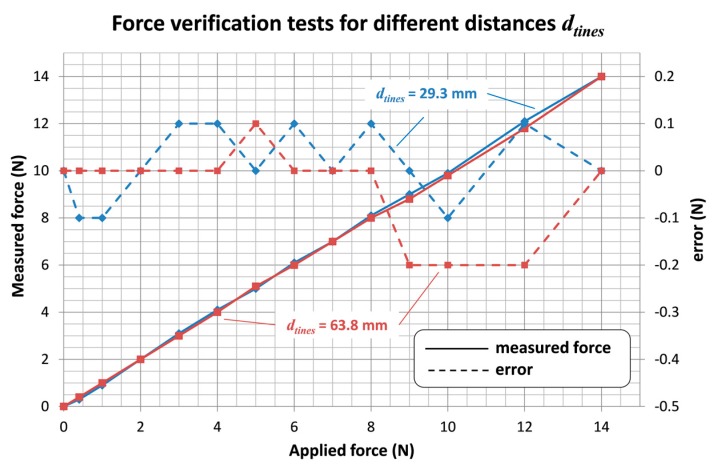
Force verification test results.

**Figure 10:**
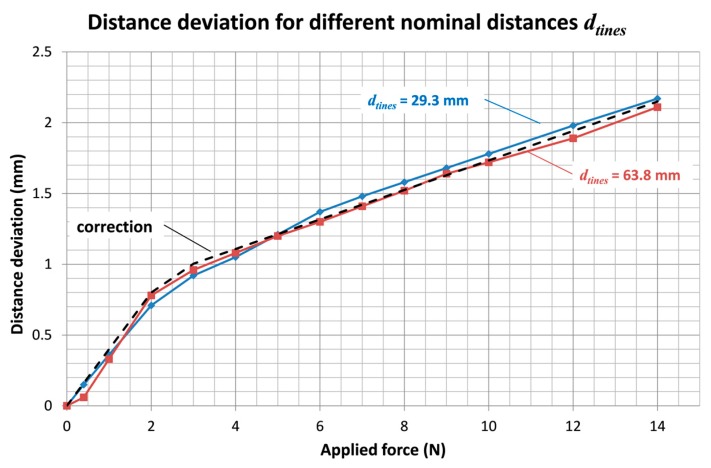
Distance deviation due to the applied separating force.

Force test results for two different values of the distance between tines are shown in Figure 9. It can be observed that the error between the force measured by the instrument and the force applied by the traction machine is equal or less than ±0.2 N. Results for the distance increase between tines due to deformation are shown in Figure 10. It can be noticed that such deviation is important, up to 2 mm for a separating force of 14 N. Therefore, the deformation phenomenon has to be considered in the distance measuring procedure. The correction curve indicated in Figure 10 has been implemented to compute the resulting distance measurement value. Thus, the distance deviation due to the applied force is lower than 0.2 mm.

**Figure 11:**
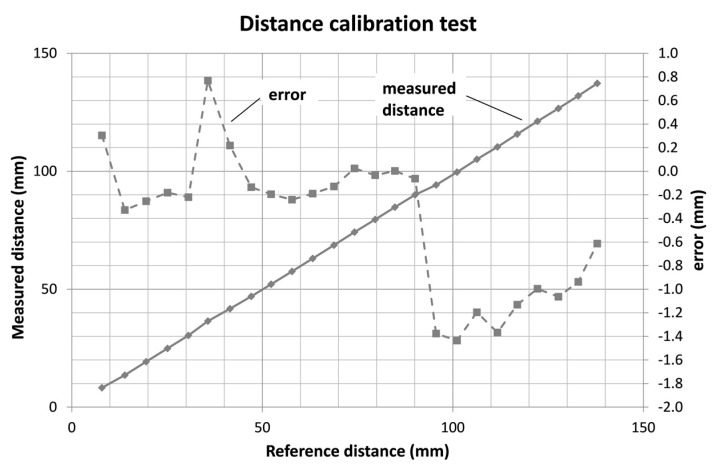
Distance verification test results.

Distance test results are shown in Figure 11. It can be observed that the error is in the range of −1.5 to +0.8 mm.

### 3.2. Functional Tests in Experimental Surgery

**Figure 12:**
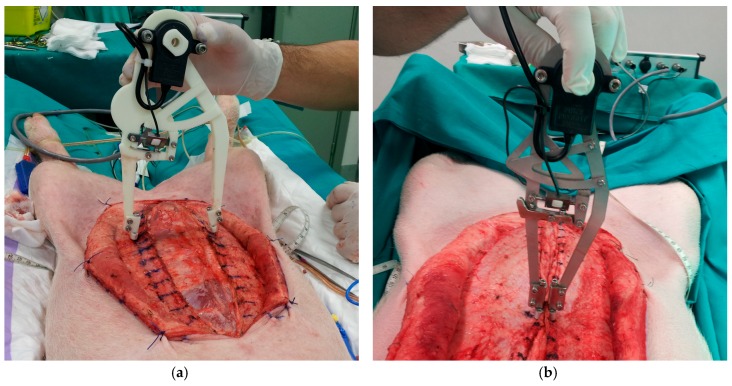
Measuring in experimental surgery with the: (**a**) 3D printed plastic prototype; (**b**) stainless steel prototype.

Instruments prototypes have been used in experimental surgical tests with pigs (see Figure 12). They have demonstrated to be very useful and provided quantitative information about the tension forces required to close different areas of the laparotomy. One of the remarkable results obtained from the measurements is that the required tensile force is always considerably higher in the supraumbilical zones of the laparotomy. Experimental tests have also allowed for establishing a preliminary relationship between the average closing tensions and the weight of the pigs.

## 4. Discussion and Conclusions

A new dynamometer has been developed to measure the tension forces and the distance between wound edges during the closure of a laparotomy. Both parameters can be easily and effectively measured simultaneously.

Since the required tension force to close an incision is an important parameter that can condition the adequate healing of the tissue, it is considered quite important to be able to evaluate it. The instrument provides objective data that can be used to help surgeons decide the most suitable closing procedure in any particular case.

Instrument accuracy has been verified and it has also been tested in experimental surgery, where it has demonstrated to be easy to use and has provided the first measurements of forces and distances in pigs.

Therefore, the developed instrument may have a high potential in clinical applications to enhance laparotomy closure procedure, since it would help surgeons to decide personalized surgical techniques.

## 5. Patents

Intellectual property rights: Patent P201630202. Instrumento para medir parámetros asociados a una incisión en un tejido y procedimiento para medir parámetros en una incisión en un tejido mediante dicho instrumento (Instrument to measure parameters associated to an incision on a tissue and procedure to measure parameters of an incision on a tissue using such instrument).
